# Laparoscopy-assisted extended right hepatectomy for giant hemorrhagic hepatic cyst mimicking biliary cystadenocarcinoma: a case report

**DOI:** 10.1186/s40792-019-0621-x

**Published:** 2019-04-11

**Authors:** Yasunari Fukuda, Tadafumi Asaoka, Hidetoshi Eguchi, Keiichiro Honma, Eiichi Morii, Yoshifumi Iwagami, Hirofumi Akita, Takehiro Noda, Kunihito Gotoh, Shogo Kobayashi, Masaki Mori, Yuichiro Doki

**Affiliations:** 1 Department of Gastroenterological Surgery, Graduate School of Medicine, Osaka University, 2-2 Yamadaoka E-2, Suita, Osaka 565-0871 Japan; 2 Department of Pathology, Graduate School of Medicine, Osaka University, Suita, Osaka Japan; 3 Department of Surgery and Science, Graduate School of Medical Sciences, Kyushu University, Fukuoka, Japan

**Keywords:** Hemorrhagic hepatic cyst, Organized hematoma, Neovascularization, Laparoscopy-assisted surgery

## Abstract

**Background:**

Hemorrhagic hepatic cysts infrequently involve several iconographic changes requiring a differential diagnosis, primarily with a cystic malignancy. We herein report a case of laparoscopy-assisted extended right hepatectomy for a giant hemorrhagic hepatic cyst with an enhancing mural nodule that was clinically suspected of being biliary cystadenocarcinoma.

**Case presentation:**

A 73-year-old woman was followed up for giant hepatic cyst occupying the right lobe of the liver. During the follow-up, an enhancing mural nodule was newly noted on computed tomography in 2016. Based on additional clinical examinations, biliary cystadenocarcinoma was undeniable, and laparoscopy-assisted extended right hepatectomy was performed for diagnostic and therapeutic purposes. She had no perioperative complications and was discharged on postoperative day 13. A histological examination of the mural nodule showed neovascularization within an organized hematoma.

**Conclusion:**

We herein report a rare case of giant hemorrhagic hepatic cyst mimicking biliary cystadenocarcinoma that was successfully treated with laparoscopy-assisted extended right hepatectomy. Laparoscopic surgery in our case was an effective procedure performed with the utmost care.

## Introduction

Intracystic hemorrhage of the liver (i.e., hemorrhagic hepatic cyst (HHC)) is an infrequent complication occurring in simple hepatic cysts with complicated iconographic characteristics (e.g.*,* a thickened cyst wall, a solid septal structure or a mural nodule) [1–5]. In particular, when enhancing solid components which are newly detected in the cyst, it is sometimes difficult to deny the presence of cystic neoplasms, such as biliary cystadenoma or cystadenocarcinoma, and surgical resection is considered for diagnostic and therapeutic purposes. When attempting a minimally invasive approach, it is of paramount importance to ensure the surgical and oncological safety [6, 7].

We herein report a case of giant HHC with an enhancing mural nodule newly arising during follow-up for hepatic giant cyst and clinically suspicious for biliary cystadenocarcinoma that was successfully treated with laparoscopy-assisted extended right hepatectomy and was pathologically diagnosed as HHC with neovascularization within an organized hematoma.

## Case presentation

A 73-year-old woman had been followed for giant hepatic cyst occupying the right lobe of the liver with a maximum diameter of 20 cm since 2005. Her medical history included a benign tumor in the transverse colon and an unruptured cerebral aneurysm. She had no remarkable family history.

**Fig. 1:**
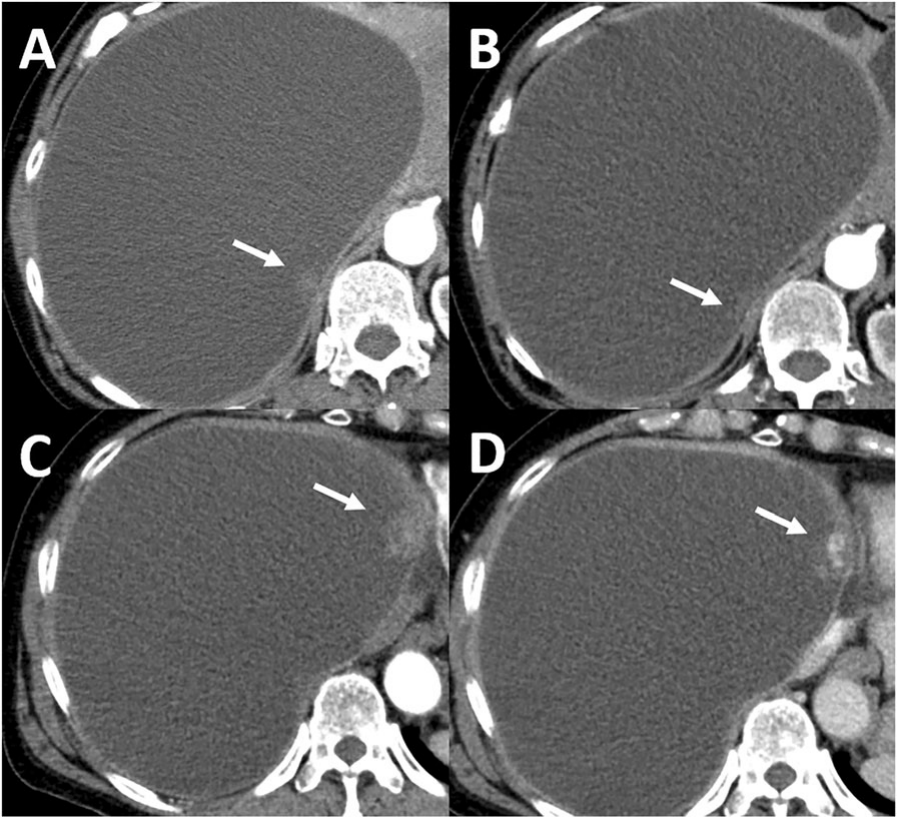
CT images of mural nodules in giant cyst. **a** Plain CT showed a dorsal unenhanced mural nodule within the cyst wall (arrow), **b** which did not change thereafter (arrow). **c** Enhanced CT showed a ventral mural nodule with weak enhancement in the early phase (arrow) and **d** with centripetal prolonged enhancement in delayed phase (arrow)

**Fig. 2:**
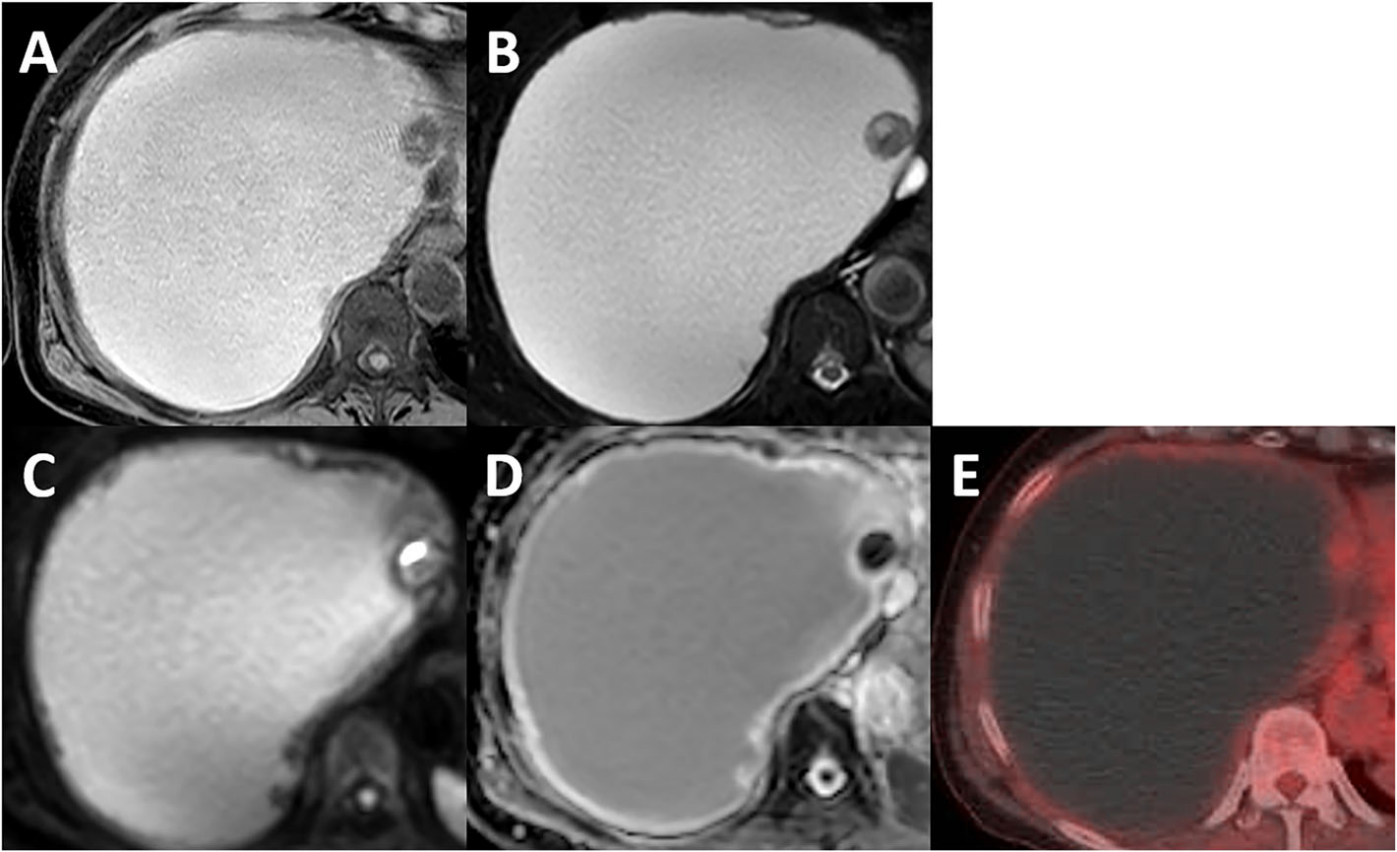
MRI and FDG-PET images of the ventral mural nodule. **a** MRI showed that the cyst content was a high-signal and the mural nodule was a low-signal on T1 W1. **b** MRI showed the cyst content was a high-signal and the mural nodule was partially high-signal on T2WI. **c** MRI showed that the mural nodule was partially strong high-signal on DWI, **d** with low ADC value (ADC_min_ 0.00 mm^2^/s, ADC_max_ 0.91 mm^2^/s, ADC_mean_ 0.6 × 10^−3^ mm^2^/s). **e** FDG-PET showed an abnormal uptake in the mural nodule with SUV-max 2.3

During the follow-up for the cyst, a dorsal unenhanced mural nodule was noted within the cyst wall on computed tomography (CT) in 2008, but no change in either the morphology or size was detected until 2016 (Fig. 1a, b). Another ventral mural nodule newly appeared in 2016. Enhanced CT showed that the ventral mural nodule was 25 mm in diameter with weak enhancement in the early phase and centripetal prolonged enhancement in delayed phase (Fig. 1c, d). Magnetic resonance imaging (MRI) showed that the cyst content had a high signal intensity on both T1- and T2-weighted imaging (WI), and the ventral nodule had low signal intensity on T1WI and partially high signal intensity on T2WI (Fig. 2a, b). In addition, the ventral nodule showed partially strong high signal intensity on diffusion-weighted imaging (DWI) (Fig. 2c) and had a low apparent diffusion coefficient (ADC) value (ADC_mean_ 0.6 × 10^−3^ mm^2^/s) (Fig. 2d). Fluorodeoxyglucose-positron emission tomography (FDG-PET) showed a weak abnormal uptake in the ventral nodule with a maximum standardized uptake value (SUV_max_) of 2.3 (Fig. 2e). Furthermore, the tumor markers CA19-9 and CEA were elevated (171 U/ml and 7 ng/ml, respectively). Considering possible malignancies such as biliary cystadenocarcinoma, she was referred to us for surgery, and surgical resection was planned for diagnostic and therapeutic purposes.

**Fig. 3:**
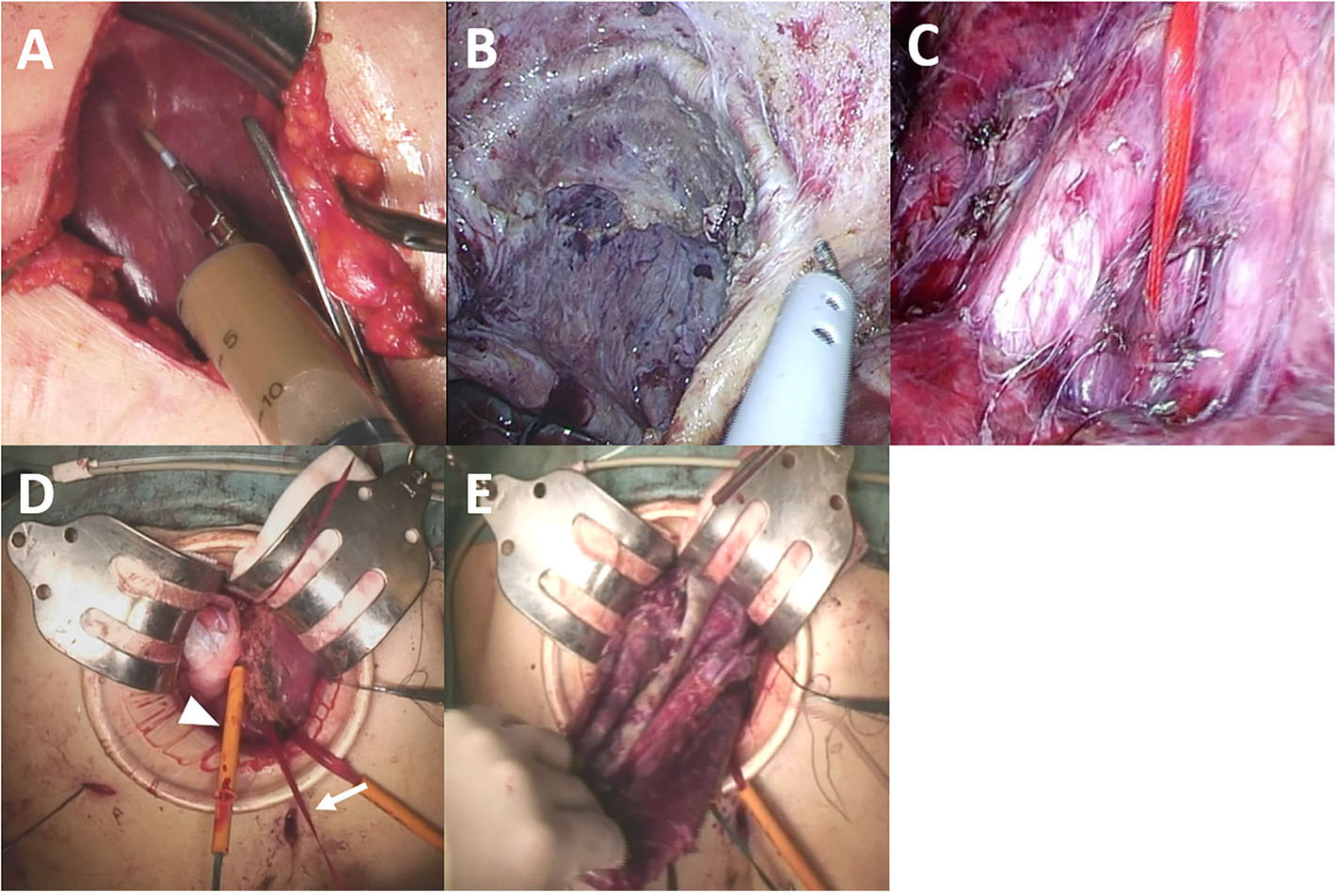
Surgical procedures. **a** The cyst content with chocolate color was trans-hepatically aspirated from a 7-cm upper midline incision. **b** The adhesiotomy between the cyst wall and right diaphragm was laparoscopically performed without rupturing the cyst wall. **c** Preparation for a hanging maneuver was performed. **d** Parenchymal division was performed under direct vision through the small laparotomy wound using a hanging maneuver (arrow) and the Pringle’s maneuver (arrowhead). **e** The specimen was carefully excised from the mini-laparotomy

We performed laparoscopy-assisted extend right hepatectomy. The surgical procedure was as follows: Under general anesthesia, a 7-cm upper midline incision was made and the cyst content was trans-hepatically aspirated (Fig. 3a). After ensuring that there were no malignant components in the cyst content by intraoperative cytology, a total of 3 L of brown serous fluid was sucked out using a 16-G elaster needle. We then continued to perform surgery as a negative result on cytology did not deny the possible existence of biliary cystadenocarcinoma [8]. The laparoscopic procedure was initiated via pneumoperitoneum with an additional four ports. First, we performed cholecystectomy and encircled the hepatoduodenal ligament (preparation for the Pringle’s maneuver). Next, laparoscopic adhesiotomy between the cyst wall and right diaphragm was performed followed by careful mobilization of the right lobe, being sure not to rupture the cyst wall (Fig. 3b). After preparing for a hanging maneuver (Fig. 3c), parenchymal division was performed under direct vision through the small laparotomy wound using a hanging maneuver and the Pringle’s maneuver (Fig. 3d) with the Glissonean pedicle approach. Finally, the specimen was carefully excised (Fig. 3e). No intraoperative complications were observed. The operative time was 673 min, and operative blood loss was 1540 ml. The postoperative clinical course was uneventful, and she left the hospital on postoperative day 13.

**Fig. 4:**
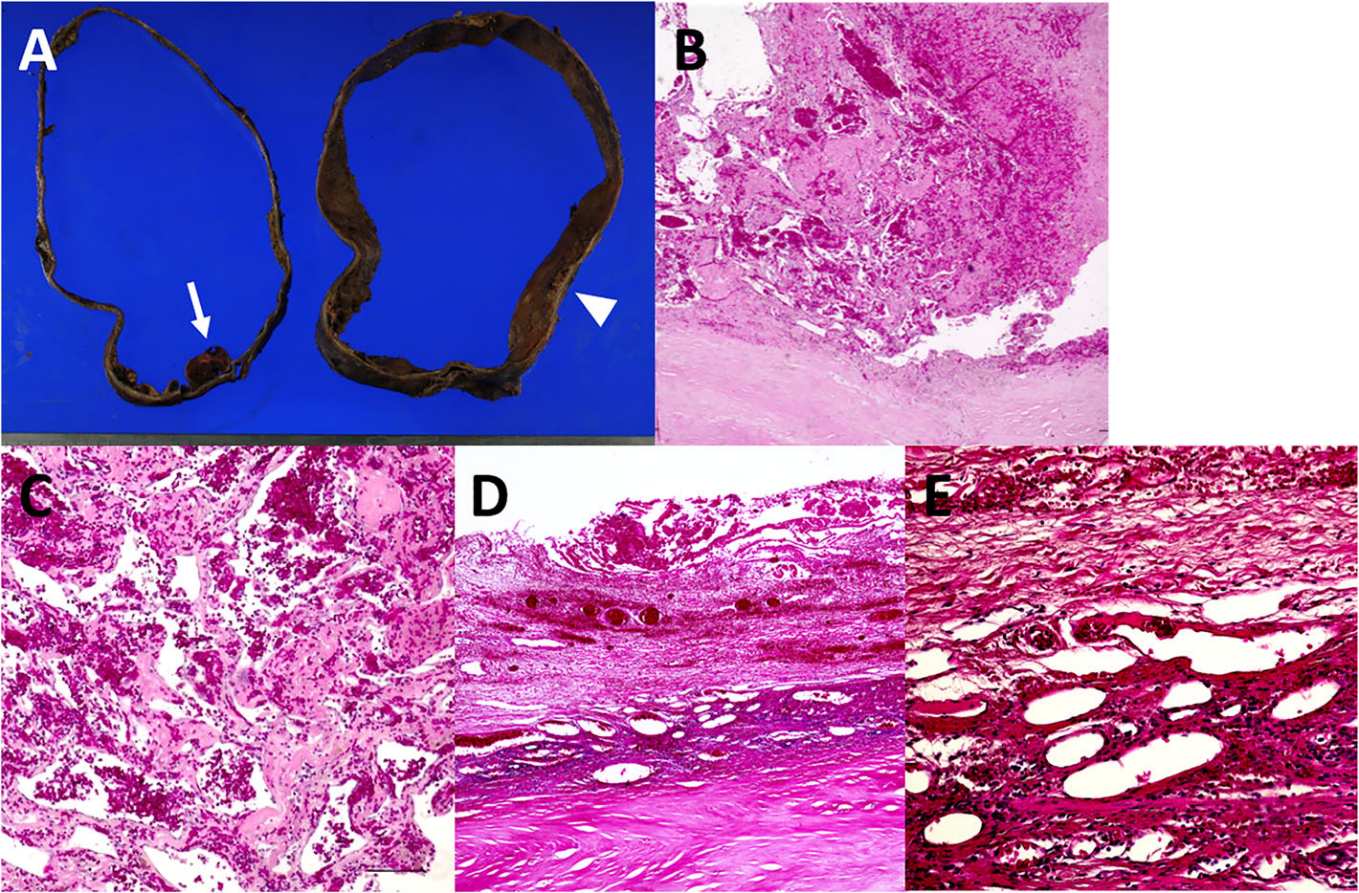
Macroscopic and microscopic findings of the resected specimen. **a** (left-hand): Macroscopically, a dark-red solid protuberance was found in the cyst wall (arrow). **a** (right-hand): Macroscopically, a thickened portion of the cyst wall where the dorsal mural nodule seemed to have originally existed was detected (arrowhead). **b**, **c** Hematoxylin and eosin staining showed that the cyst wall mainly consisted of thick fibrous stroma and the ventral mural nodule consisted of several sizes of blood-filled vascular cavities surrounded by endothelial cells within the organized hematoma (original magnification: **b** × 40, **c** × 100). **d**, **e** Hematoxylin and eosin staining showed that the thickened portion of the wall consisted of malformed veins of various sizes within the calcified hematoma (original magnification: **d** × 40, **e** × 200)

**Fig. 5:**
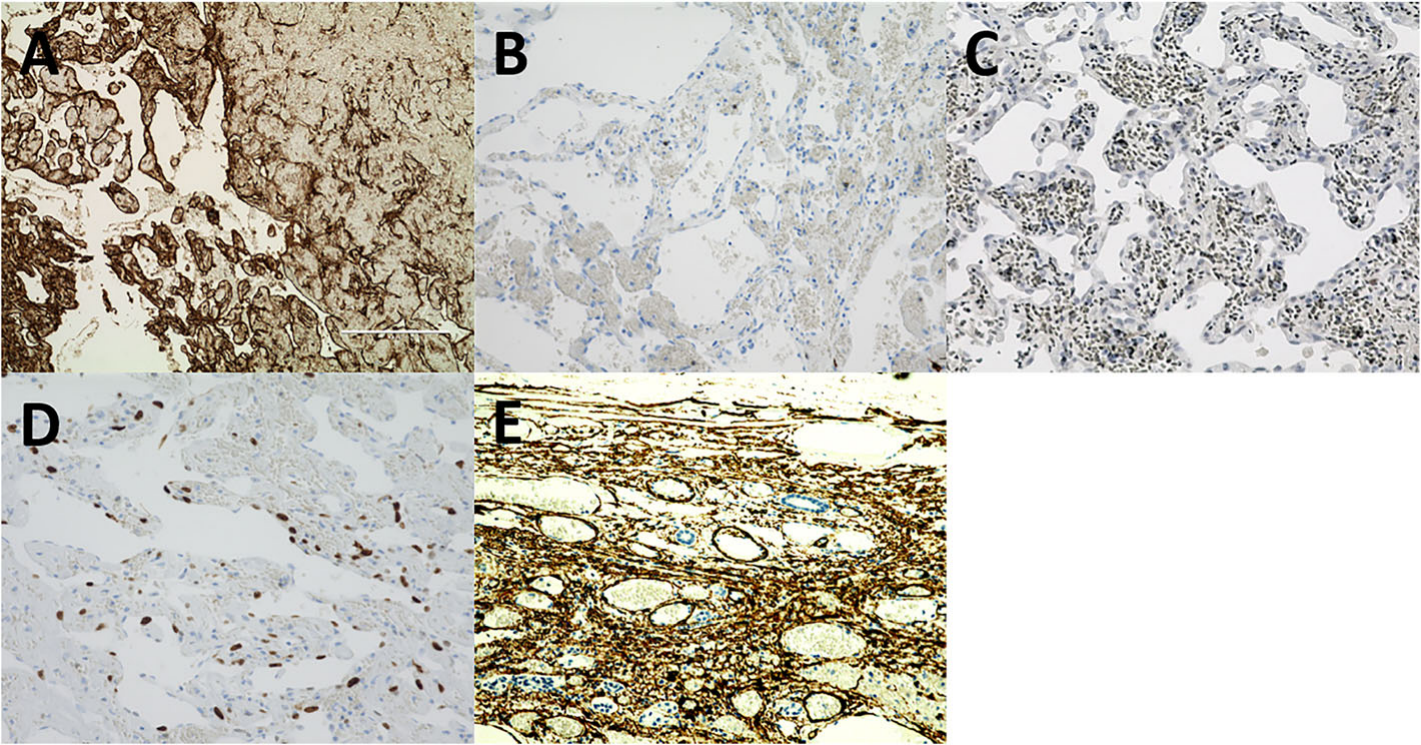
Immunohistochemical staining. Immunohistochemical staining of ventral mural nodule was **a** positive for CD31 (original magnification × 40) and **b** negative for αSMA and **c** MDM2 (original magnification × 200). **d** The Ki-67 labeling index was 19.0% (original magnification × 200). **e** Immunohistochemical staining of the thickened portion of the cyst wall was positive for αSMA (original magnification × 200)

Macroscopically, there was a dark-red solid protuberance in the cyst wall (left side of Fig. 4a), consistent with the ventral enhancing mural nodule. A histological examination showed that the cyst wall mainly consisted of thick fibrous stroma and the mural nodule consisted of blood-filled dilated vessels lined by endothelial cells within the organized hematoma, mimicking cavernous hemangioma (Fig. 4b, c). Immunostaining of the dilated vessels showed positivity for CD31 (Fig. 5a) but negativity for α-smooth muscle actin (α-SMA) (Fig. 5b) and Elastica van Gieson (EVG) (data not shown), and murine double minute (MDM) 2 (Fig. 5c) and p53 (data not shown), markers for liver angiosarcoma. The Ki-67 labeling index was 19.0% (Fig. 5d). Therefore, we pathologically diagnosed the patient with HHC with neovascularization within the organized hematoma. On the other hand, because the dorsal mural nodule could not be detected macroscopically, we evaluated a thickened portion of the cyst wall where the dorsal mural nodule seemed to have originally existed (right side of Fig. 4a). The thickened portion consisted of malformed veins of various sizes (Fig. 4d, e) within a calcified hematoma. These veins were immunohistochemically positive for α-SMA (Fig. 5e) and EVG (data not shown). She has survived for 2 years without any evidence of recurrence after surgery.

## Discussion

We encountered a case of successful laparoscopic surgery performed for giant HHC with an enhancing mural nodule that was clinically suspected of being biliary cystadenocarcinoma.

Laparoscopic liver resection (LLR) has rapidly gained acceptance over the past decade as a viable treatment for several malignant and benign hepatic diseases, given its prominent advantages over an open approach with regard to post-surgical pain relief, better cosmetic results, and a shorter hospital stay due to the smaller surgical wound and less bleeding. The recent international consensus is that minor LLR is a safe and feasible procedure that is superior to an open approach for select patients; in contrast, major LLR remains a technically demanding procedure, and its safety, feasibility and preliminary evidence are still limited [7].

At our institute, we have expanded the indication for LLR as we accumulate experience, and laparoscopic-assisted extended right hepatectomy was completed with sufficient surgical and oncological safety in the present case. Remarkable surgical techniques applied in this case included the shrinkage of the cyst and the hanging maneuver technique. First, we carefully removed the cyst contents without any spillage because shrinkage of the cyst was necessary in order to create an intraabdominal working space. We were subsequently able to mobilize the right lobe of the liver laparoscopically. Second, the hanging maneuver technique was effective for ensuring the safe performance of parenchymal division through the small laparotomy wound (7 cm), as this technique makes it possible to pull up the liver so that the resection line comes just beneath the laparotomy wound [9].

Previous studies have discussed the imaging characteristics of HHCs; however, they still remain challenging to describe [1–5]. Very recently, Kohno et al. summarized the imaging characteristics of HHCs with enhancing mural nodules [5]. In all the resected cases in the literature, the nodules were histologically diagnosed with neovascularization within organized hematoma, as was observed in our case. As they mentioned, the ventral enhancing mural nodule in our series showed persistent centripetal filling on delayed CT images and central high signal intensity on T2WI. In addition, the calcification of the cyst wall was found close to the dorsal mural nodule. However, unlike their report, the ventral mural nodule in the present case demonstrated a weak but abnormal uptake on FDG-PET. This abnormal uptake on PDG-PET may have been associated with the high Ki-67 value. Previous studies have shown that the SUV_max_ on FDG-PET was correlated with the Ki-67 index of various tumors [10, 11]. Considering that the ventral mural nodule rapidly grew within a year, it is conceivable that the nodule included highly proliferating endothelial cells. Moreover, we found a high signal intensity area on DWI with a low ADC value on MRI in the ventral mural nodule, and this was retrospectively considered to be consistent with organized hematoma. However, these MRI findings were also observed in biliary cystadenocarcinoma, which led to the preoperative misdiagnosis.

Because HHC is a rare and heterogeneous entity, its cause and pathogenesis have not been fully discussed. In the present case, we hypothesize that giant HHC was formed as a result of repeated rupture of venous malformation and that neovascularization developed reactively in association with the thrombus undergoing organization. Hepatic hemangioma with cyst formation has been also reported as a rare condition [12, 13], and our case bore some similarities to this entity. However, the dilated vessels in the ventral mural nodule were immunohistochemically negative for αSMA and EVG, indicating reactive angiogenesis mimicking cavernous hemangioma.

## Conclusions

In conclusion, we reported a rare case of HHC mimicking biliary cystadenocarcinoma successfully treated with laparoscopy-assisted extended right hepatectomy. When a new enhancing solid nodule appears within HHC, it is difficult to rule out cystic neoplasms preoperatively in many cases. However, we should bear in mind the possible presence of HHC with neovascularization within the organized hematoma, and laparoscopic surgery can be a diagnostic and therapeutic option if technically possible and performed with the utmost surgical and oncological care.

## Abbreviations

ADC: Apparent diffusion coefficient
CT: Computed tomography
DWI: Diffusion-weighted imaging
EVG: Elastica van Gieson
FDG-PET: Fluorodeoxyglucose-positron emission tomography
HHC: Hemorrhagic hepatic cyst
LLR: Laparoscopic liver resection
MDM: Murine double minute
MRI: Magnetic resonance imaging
SMA: Smooth muscle actin
SUV: Standardized uptake value
T1 and T2WI: T1- and T2-weighted imaging
